# Intestinal obstruction due to idiopathic mesenteric phlebosclerosis colitis: A case report

**DOI:** 10.3389/fsurg.2022.969154

**Published:** 2022-08-17

**Authors:** Jiaqi Shan, Fangci Chen, Panpan Yu

**Affiliations:** ^1^ The Fourth School of Clinical Medicine, Zhejiang Chinese Medical University, Hangzhou, China; ^2^ Department of Gastrointestinal Surgery, Hangzhou First People’s Hospital, The Affiliated Hospital of Medical School of Zhejiang University, Hangzhou, China

**Keywords:** idiopathic mesenteric phlebosclerosis colitis, phlebosclerotic colitis, intestinal obstruction, surgical procedure, Chinese herbal medicine

## Abstract

**Introduction:**

Idiopathic mesenteric phlebosclerosis colitis (IMP) is a rare condition that impairs colonic venous blood return owing to mesenteric venous sclerosis and fibrosis. At present, many studies have suggested that long-term intake of Chinese herbal medicines is associated with its pathogenesis. IMP has no characteristic clinical manifestations, and most patients with IMP present with acute intestinal obstruction. As a rare disease, the etiology, pathogenesis, pathophysiology, and treatment of IMP are being explored and studied.

**Case Description:**

A 60-year-old Chinese male patient with IMP was admitted to our hospital for acute intestinal obstruction, received subtotal colectomy and ileostomy after 10 days of ineffective conservative treatment, and was discharged after postoperative supportive treatment for 1 month.

**Conclusion:**

There are many causes of intestinal obstruction, and we report a relatively rare one. After failure of conservative treatment, it is necessary to surgically resect part of the diseased bowel.

## Introduction

Idiopathic mesenteric phlebosclerosis colitis (IMP), also known as phlebosclerotic colitis, is a rare ischemic colitis with an incidence of 0.01/100,000 individuals in Japan. It occurs almost exclusively in Asian populations, and it is most prevalent in Japan and China. The etiology and pathogenesis of IMP have not been clarified. Studies have associated this disease with the long-term use of Chinese herbal medicines, containing *Gardenia jasminoides*. Although its clinical manifestations are nonspecific, IMP presents with distinct imaging findings. The treatment options for IMP include conservative management and surgery.

This study reports the case of a patient who presented with intestinal obstruction and was preoperatively diagnosed with IMP. The patient was successfully cured and discharged after undergoing surgery. Ethical review and approval were not required for the study on human participants in accordance with the local legislation and institutional requirements. The patient provided written informed consent to participate in this study. Written informed consent was obtained from the individual for the publication of any potentially identifiable images or data included in this article.

## Case report

**Figure 1:**
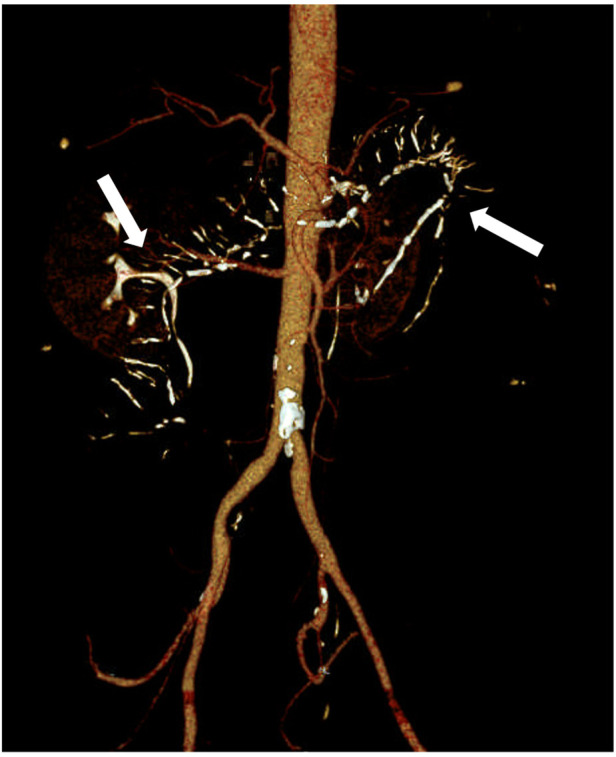
Abdominal vascular computed tomography (CT) angiography revealed atherosclerotic changes in the lower abdominal aorta, bilateral common iliac arteries, and internal iliac arteries, with corresponding mild stenosis and extensive calcification of the venous network in the ascending and transverse parts of the colon (arrow).

**Figure 2:**
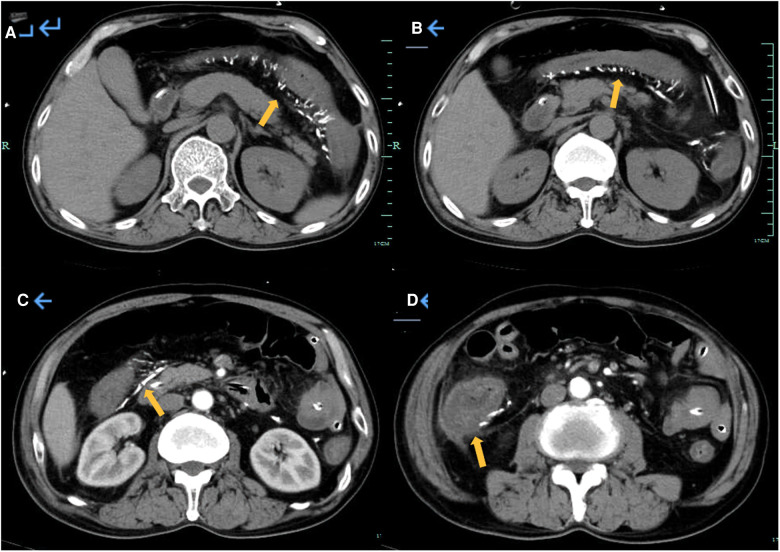
Abdominal CT and enhanced CT revealed extensive calcification of the venous network in the ascending colon and transverse colon, with edema and thickening of the intestinal wall and small intestinal obstruction.

A 60-year-old Chinese male patient complaining of abdominal pain for >3 days and vomiting for >2 days was admitted to our hospital in February 2022. Three days prior, the patient had experienced abdominal distension and discomfort in the right lower quadrant, relieved by eating. The pain recurred 1 day later and was accompanied by nausea, vomiting, and constipation. The patient was admitted with a primary working impression of intestinal obstruction. He had diarrhea for more than 6 years, passing loose stools twice a day on average. The patient also had medical history of syphilis. The patient had been using Chinese herbal medicine, specifically Zhizhonghe *Cortex acanthopanacis* wine, for more than 20 years at a dose of 300 g per day. He had an unremarkable family history. On examination, the patient had a distended abdomen with tenderness at the right lower quadrant and no rebound pain. Laboratory examination showed a peripheral white blood cell count of 13.4 × 10^9^/L and a C-reactive protein level of 194.8 mg/L. Computed tomography (CT) angiography revealed atherosclerotic changes with mild luminal stenosis in the lower segment of the abdominal aorta, left and right common iliac arteries, and left and right internal iliac arteries (Figure 1). Extensive calcification of the ascending and transverse colonic venous networks was observed. The bowel wall was edematous and thickened, and small bowel obstruction was observed (Figure 2). Based on these findings, IMP was considered.

On admission, the patient received symptomatic and supportive treatment, consisting of fasting, gastrointestinal decompression, acid inhibition, gastric protection, fluid replacement, parenteral nutrition, and anti-infection treatment with 2 g of cefotaxime sodium every 8 h. After 10 days of conservative treatment, no significant improvement in the patient's symptoms was noted. Abdominal CT revealed intestinal wall swelling with encapsulated effusion in the paracolic gutter of the adjacent fat colon. These findings suggested intestinal perforation, which was an indication for surgery.

**Figure 3:**
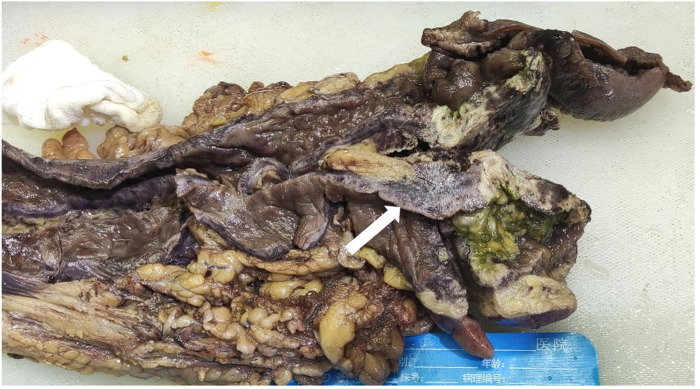
Macroscopically, the colonic mucosa appeared dark purple, and the colonic wall was significantly thickened (arrow).

**Figure 4:**
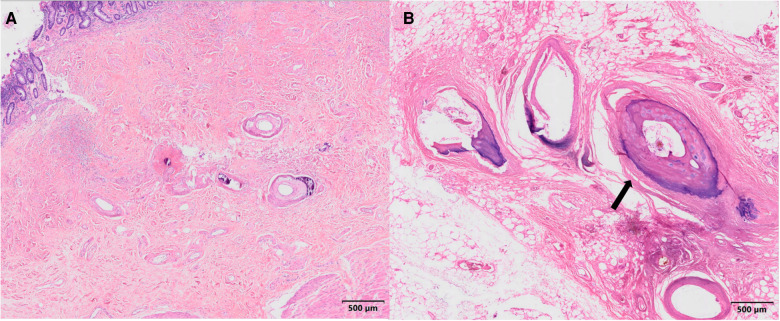
(**A**) Histological microscopy showing epithelial detachment in the lamina propria of the intestinal mucosa, local vascular slide, interstitial collagenization, and mucosal muscle atrophy in the area. The vascular wall in the submucosa was extensively thickened and narrowed with slide, local calcification, and interstitial collagenization (hematoxylin and eosin staining). (**B**) Arrow: Extensive stenosis, occlusion with degeneration, calcification, and ossification of thick-walled vessels on the serosal side (hematoxylin and eosin staining).

After excluding contraindications to the procedure, laparoscopic exploration was performed on February 25. Intraoperatively, the ascending and descending colon tubes were violaceous, and poor peristalsis was noted. The intestinal wall of the ascending colon was necrotic, thin, and densely adherent to the lateral peritoneum, forming a pus cavity. There were no palpable masses in the intra-abdominal wall, enlarged lymph nodes at the mesenteric root, or ascites in the abdominal cavity. Given the difficulty of separating the ileocecal region, laparotomy with subtotal colectomy and ileostomy were performed. IMP was confirmed by postoperative pathological findings (Figures 3, 4).

Postoperatively, the patient continued receiving cefotaxime sodium for protection against infections, supplemented by hemostatic and analgesic incision dressing change and other supportive means of treatment. The patient fully recovered and was discharged on March 24.

## Discussion

Here, we reported the case of a 60-year-old male patient who used Chinese herbal medicine for longer than 20 years, developed IMP and acute intestinal obstruction, and was successfully treated with surgery and supportive postoperative care. IMP is a rare clinical condition. With more than 200 reported cases, IMP almost exclusively affects the Asian population, and it is most prevalent in Japan and China. Only few cases have been reported outside Asia (1, 2). According to previous studies, IMP more commonly affects middle-aged and elderly men (3).

This disease was first reported in Japan by Koyama et al*.* in 1991. It was termed “phlebosclerotic colitis” (4) by Yao et al. in 2000 and “idiopathic mesenteric phlebosclerosis” by Iwashita et al. in 2003 (5).

The etiology and pathogenesis of IMP have not been clarified. Recent studies have associated IMP with the chronic intake of biochemical substances and toxins (6). The long-term use of Chinese herbal medicines containing geniposide was related to this disease (7, 8). The patient in the present study had history of long-term intake of Zhizhonghe *Cortex acanthopanacis* wine, consisting of white wine, honey wine, *Gardenia jasminoides* Ellis, and *Cortex acanthopanacis*. These ingredients were similar to those noted in previous reports on the association between IMP and Chinese herbal medicine use. The aqueous decoction of *Gardenia jasminoides* contains large amounts of geniposide, which is metabolized into genipin by β-glucosidase, produced by proximal colonobacteria, and reacts with proteins in the mesenteric venous plasma. The gradual accumulation of collagen under the mucosa is followed by the development of a hyperplastic endomysium in the veins, accompanied by fibrosis, resulting in venous obstruction (9). However, Chen et al. found that only four of 25 Chinese patients with IMP had history of long-term use of traditional Chinese medicinal materials and medicinal liquor (10). In a study by Sheung et al. involving 29 patients with venous sclerosing colitis, only six used various herbal medicines, and a significant association between herbal medicines and IMP was not established (11). According to a systematic review of 240 reports by Wang et al., 78.7% of patients had history of herbal medicine intake (12). A survey in Japan showed that 147 of 222 patients used traditional Chinese medicine (13). There have also been reports of mother and daughter pairs (14) and couples (15), who all used Chinese herbal medicines for a long time and developed the disease. The risk factors for IMP include diabetes, cirrhosis, portal hypertension, and hypercoagulable states.

IMP is pathologically characterized by mucosal ulceration, epithelial ischemic atrophy of the intestinal wall, extensive fibrosis and hyalinization of the submucosa (16), and marked calcification within the venous wall narrowed by non-inflammatory fibrosis (11). The clinical signs of IMP are nonspecific and include chronic abdominal pain, diarrhea, and anemia. Acute ischemia aggravates the symptoms, leading to intestinal obstruction and intestinal perforation. There have been case reports of IMP, accompanied by precancerous lesions of colonic polyps or colon cancer. Thus, close follow up during conservative management is indicated in patients with IMP to prevent disease aggravation and carcinogenesis. However, the relationship between IMP and colon cancer requires further investigation.

Various methods have been used to examine IMP. Diagnostic modalities include radiography, barium enema, angiography, colonoscopy, and CT. IMP has characteristic imaging features, and CT accurately detects these findings. The main CT findings of IMP included thickening of the affected colonic wall and linear calcification of the mesenteric vein. Typical endoscopic findings include a dark violaceous or blue intestinal wall in the diseased segment of the colon (17). This is likely caused by chronic congestion with ischemia or toxin contamination of the intestinal mucosa. It has also been related to the dark blue color of prednisone upon reacting with amino acids or proteins. In addition, digital subtraction angiography, CT angiography, and ultrasound endoscopy are useful diagnostic tools for IMP.

The diagnosis of IMP is based primarily on imaging findings, clinical symptoms, and history of relevant drug intake. CT findings of curvilinear mesenteric vein calcification confirm the diagnosis, and CT imaging is also used to assess the severity of the disease.

The primary treatment strategy for IMP is conservative. Ingestion of Chinese herbal medicines should be stopped. Symptomatic and supportive treatments, including intestinal rest, improvement of microcirculation, and infection prevention, are advised. Total or subtotal colectomy is recommended when there is a wide range of lesions (lesions involving the rectum), severe complications such as intestinal obstruction and intestinal perforation, or recurrent IMP with mild symptoms. With proper treatment, IMP has a favorable prognosis.

## Conclusion

IMP is a rare disease with unknown etiology, and further studies are needed to explore the specific risk factors and etiology of IMP in order to reduce the incidence and identify high-risk groups. In this paper, we report a case of intestinal obstruction caused by IMP, and we discuss the necessity to perform surgical resection of the diseased part of the bowel when conservative treatment has not been effective.

## Data Availability

The raw data supporting the conclusions of this article will be made available by the authors, without undue reservation.
